# Biological Activity of Fermented Plant Extracts for Potential Dermal Applications

**DOI:** 10.3390/pharmaceutics15122775

**Published:** 2023-12-14

**Authors:** Anna Herman, Andrzej Przemysław Herman

**Affiliations:** 1 Chair of Drug and Cosmetics Biotechnology, Faculty of Chemistry, Warsaw University of Technology, Koszykowa 75 Street, 00-662 Warsaw, Poland; 2 Department of Genetic Engineering, The Kielanowski Institute of Animal Physiology and Nutrition, Polish Academy of Sciences, Instytucka 3 Street, 05-110 Jabłonna, Poland; a.herman@ifzz.pl

**Keywords:** fermented plant extract, antioxidant activity, anti-inflammatory activity, anti-melanogenic activity, wound healing activity

## Abstract

Fermented plant extracts (FPEs) are functional liquids formed as a result of the fermentation of fresh plants by microorganisms, mainly bacteria and fungi. The appropriate selection of plants, microorganism strains, and conditions under which the fermentation process is carried out is very important in terms of obtaining a suitable matrix of biologically active compounds with different biological properties. The purpose of this review is to provide verified data on the current knowledge acquired regarding the biological activity of FPEs for cosmetic use and dermal applications. The antioxidant, antimicrobial, anti-inflammatory, anti-melanogenic, and wound-healing activity of FPEs, as well as their potential dermal applications, will be described.

## 1. Introduction

Fermented plant extracts (FPEs) are functional liquids formed as a result of the fermentation of fresh plants by microorganisms, mainly bacteria and fungi. The fermentation process enhances the biological activities of the substrate by converting high-molecular compounds into low-molecular structures, making fermented raw materials more compatible compared to unfermented ones [1]. Fermentation depends largely on the selection of the microorganism strain used and the conditions under which it is carried out. The appropriate choice of microorganisms and plants is critical to obtaining the desired matrix of biologically active compounds. The structural breakdown of plant cell walls and hydrolysis activity of the bacteria/fungi during fermentation increased polyphenols, flavonoids, organic acids, proteins, ceramides, amino acids, biological enzymes, and antioxidants in the fermentation medium [2,3]. Moreover, the product obtained after plant fermentation shows increased biological effectiveness and bioavailability with decreased cytotoxicity [2]. The fermentation of *Camellia sinensis* with mixture of *Lactobacillus rhamnosus, Lactobacillus plantarum*, and *Saccharomyces cerevisiae* [4], blueberry fruits with lactic acid bacteria (*Lactobacillus plantarum, Lactobacillus fermentum*) [5], and black tea with the kombucha [6] confirm the presence of phenolic compounds in fermentation medium. Moreover, fermentation time affects the content of bioactive compounds in bioferment. A fermentation medium with kombucha yerba mate extract showed that after 14 and 21 days of fermentation, the content of bioactive compounds, mainly polyphenols (chlorogenic acid, caffeoyl derivatives), as well as xanthines and flavonoids, may indicate a biological potential of fermented plant extract for dermatological use [7]. Recently, polyphenols have gained much more attention, owing to their possible beneficial implications in human health, such as in the treatment and prevention of cancer, cardiovascular disease, aging-associated mental deterioration, and neurodegeneration [8]. It was shown that fermented *Magnolia denudata* flower petal extract with *Pediococcus acidilactici* KCCM 11614 was higher than that of non-fermented plant extract against human gastric adenocarcinoma cell line (AGS), human cervical carcinoma (HeLa), and human colorectal carcinoma (LoVo) cells [9]. Also, Jo et al. [10] showed that ginseng extract fermented by *Aspergillus usamii* had better anticancerous activity against human hepatoma (HepG2), AGS, and human colon adenocarcinoma (DLD-1) cells in comparison to the non-fermented extract. Fermented *Rhus verniciflua* stem bark extracts showed anticancer activity in a colon cancer cell line HCT-116 and the ability to induce senescence or apoptosis and inhibit the hedgehog pathway [11]. The fermented *Ophiopogon japonicas* extract with *Cordyceps militaris* (first fermentation for 10 days), *Lactobacillus plantarum, Enterococcus faecium*, and *Bifidobacterium longum* (second fermentation for 2 days) can prevent cardiovascular diseases associated with the proliferation and migration of vascular smooth muscle cells (VSMCs) [12]. In turn, fermented *Withania somnifera, Emblica officinalis*, and *Bacopa monnieri* extract with *Beauveria bassiana* ATCC 7159 reduced chick embryo chorioallantoic membrane (CAM) vascularization, suggesting its anti-angiogenic potency [13]. This result poses application potential and may be used in many disorders due to uncontrolled vessel proliferation, such as atherosclerosis, diabetic retinopathy, rheumatoid arthritis, psoriasis, keratitis, glaucoma, and solid tumor development. Moreover, the fermented *Withania somnifera, Emblica officinalis*, and *Bacopa monnieri* extract were formulated for a nutraceutical anti-angiogenic treatment of age-related macular degeneration and commercialized in an oral form named Ethnodyne-VisioTM (Ethnodyne, France). The fermentation of *Ginkgo biloba* with *Aspergillus niger* enhances its neuro-protective role via antioxidant, anti-apoptotic and anti-inflammatory activities, leading to the amelioration of the stress hormones (catecholamines, epinephrine, norepinephrine, dopamine) compared to the non-fermented *Ginkgo biloba* leaf extract [14]. Fermented *Carica papaya* with yeast (commercialized as Immun’Age, Osato Research Institute, Gifu, Japan) significantly decreased 8-hydroxy-2′-deoxyguanosine (oxidative stress marker) in urine patients with Alzheimer’s disease [15] as well as reduced experimental ischemia-reperfusion-induced cerebral damage [16]. FPE, a rich source of bioactive compounds with various biological activities, may also gain interest in the cosmetics and pharmaceutical industries.

The purpose of this review is to provide verified data on the current knowledge acquired biological activity of fermented plant products for dermal applications.

## 2. Methods

### 2.1. Search Strategy

The PubMed, Scopus, and Google Scholar databases were used to search articles published from 2010 to 2023. Search terms included ‘fermented plant extract’, ‘fermented plant product’, ‘fermented plant extract biological activity’, and ‘fermented plant extract in dermal usage’. References from reviews regarding fermented plant products were also searched for additional articles and case reports. A manual search was also performed based on citations in the published literature.

### 2.2. Inclusion and Exclusion Criteria

All publications describing the biological activities of FPEs related to their dermal application have been included in this study. Other applications (e.g., food industry) of FPE than dermatology were excluded from this study. Publications in languages other than English were also excluded. Finally, 37 articles that meet the criteria were used for the review.

## 3. Biological Activity of Fermented Plant Extract for Dermal Applications

**Figure 1:**
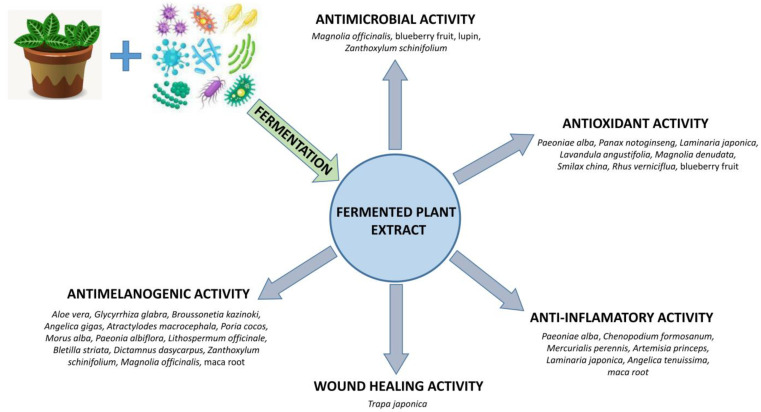
Biological activity of FPE for dermal applications.

Some research showed that fermentation may improve the biological activities of the plant and enhance the production of bioactive compounds [3]. Fermented plant products showed varied biological activity, including antimicrobial, antioxidant, anti-inflammatory, anti-melanogenic, and wound-healing activity (Figure 1). FPE activities depend on the selection of microorganism strain used in the fermentation process in order to obtain a mixture of compounds characterized by the desired biological activity (Table 1).

### 3.1. Antimicrobial Activity

Fermented Magnolia officinalis extracts with Aspergillus niger showed greater antibacterial activity against tested strains (*Escherichia coli*, *Staphylococcus aureus*, *Bacillus subtilis*, *Staphylococcus epidermidis*, *Propionibacterium acnes*, *Epidermophyton floccosum*, methicillin-resistant *Staphylococcus aureus*) and significantly increased 8–20-fold compared with that of the unfermented extracts [30]. The antibacterial activities against various bacterial strains, including methicillin-resistant *Staphylococcus aureus* (MRSA), were due to the enhancement of concentrations of antimicrobial compounds in the fermented Magnolia officinalis extracts (e.g., chlorogenic acid, magnolol, honokiol, and quercetin) and the production of new compounds with antimicrobial activity (e.g., catechin and ferulic acid) by *Aspergillus niger* fermentation [30]. Fermented *Zanthoxylum schinifolium* extract with *Lactobacillus rhamnosus* A6-5 showed greater antibacterial activity against *Propionibacterium acnes* and *Staphylococcus epidermidis* than the raw extract [36]. Fermented blueberry fruit extract with selected probiotic bacteria (*Bacillus amyloliquefaciens* and *Lactobacillus brevis*) and yeast (*Starmerella bombicola*) isolated from fermented starfish showed antibacterial activity against *Brevibacterium linens*, *Propionibacterium acnes*, *Bacillus cereus*, and *Staphylococcus* epidermidis [21]. Moreover, *B. amyloliquefaciens* and *S. bombicola* fermented blueberry fruit extracts appeared to possess a higher antimicrobial activity against skin bacteria and lower minimum inhibitory concentration (MIC) and minimum bactericidal concentration (MBC) values than *L. brevis* fermented extracts. Also, selenium nanoparticles synthesized by fermented aqueous extract of lupin by *Aspergillus oryzae* (ability to reduce selenium ion in the presence of gamma rays) were active towards *Acinetobacter calcoaceticus*, *Staphylococcus aureus*, *Candida albicans* and *Aspergillus flavus* [28]. Only a few studies show that FPE has antibacterial and antifungal properties [21,28,30,36]. Unfortunately, their antimicrobial mechanism of action remains unknown.

### 3.2. Antioxidant Activity

Fermentation also can improve the antioxidant activity of plant extracts associated with increased phytochemicals, mainly by polyphenols, antioxidant polysaccharides, and antioxidant peptides produced by microbial hydrolysis or biotransformation [37]. Fermented *Paeoniae alba* extract with plant-derived *Lactobacillus brevis* 174A significantly elevated the total phenolic content, reduced intracellular reactive oxygen species (ROS) levels, and inhibited the nitric oxide (NO) release [32]. The fermented *Panax notoginseng* polysaccharides (FPNP) with *Saccharomyces cerevisiae* CGMCC 17452 protect against the oxidative damage of collagen and elastin induced by H_2_O_2_ via TGF-β/Smad signaling pathway in human fibroblast cells [33]. Moreover, FPNP decreases ROS and malondialdehyde (MDA) contents, reversed the reduction in the antioxidant activity and the expression of the antioxidant enzyme catalase (CAT), glutathione peroxidase (GSH-Px), and superoxide dismutase (SOD) induced by H_2_O_2_. Furthermore, the upregulation in the expression of transforming growth factor-β (TGF-β), Smad2/3, and the downregulation in the expression of Smad7 in FPNP-treated groups showed that through the activation of the TGF-β/Smad signaling pathway, FPNP inhibited H_2_O_2_-induced collagen and elastin-injury in human fibroblast cells. Fermented *Laminaria japonica* extract with *Saccharomyces cerevisiae* has stronger antioxidant activity than unfermented *Laminaria japonica* extract [26]. Fermented *Laminaria japonica* extract possesses strong free radical scavenging ability via increases in the synthesis of antioxidant enzymes in human immortalized epidermal keratinocytes (HaCaT) exposed to UVB radiation. Also, *Lavandula angustifolia* extract fermented with *Pediococcus pentosaceus* DK1 showed higher inhibition of ROS generation than those treated with non-fermented extract [27].

The antioxidative effect of *Magnolia denudata* flower petal extract fermented by *Pediococcus acidilactici* KCCM 11614 was threefold higher than that of the (non-fermented) control [9]. Moreover, the 1,1-diphenyl-2-picryl-hydrazyl (DPPH) radical scavenging activity of fermented magnolia increased from 85.1% to 91.4% depending on the fermentation time, while those of the non-fermented plant extract were not significantly different. Fermented *Rhus verniciflua* bark methanol extract showed the highest DPPH radical-scavenging activity for ethyl acetate fraction and then beta hydroxy acid (BHA) and butylated hydroxytoluene (BHT) as control [11]. Fermented extract from *Smilax china* leaves with mixtures of *Lactobacillus bulgaricus* and *Lactobacillus reuteri* showed DPPH scavenging activity at 0.0625%, the same as ascorbic acid, and the maximum DPPH scavenging activity (92.44%) at 1% [34]. The 2, 2’-azino-bis (3-ethylbenzthiazoline-6-sulphonic acid (ABTS) and DPPH assays showed significant scavenging activity in fermented blueberry extract with *Bacillus amyloliquefaciens, Lactobacillus brevis*, and *Starmerella bombicola* [21]. Fermented for 28 days, green coffee beans with kombucha characterized the highest antioxidant capacity and may be a valuable source of bioactive substances used in cosmetic and dermatological products [25].

### 3.3. Anti-Inflammatory Activity

A large number of herbal products possess active constituents that retard the key steps of the inflammation pathway (nuclear factor kappa B (NF-κβ), lipoxygenase (LOX), and cyclooxygenase (COX)) [38]. The antioxidant compounds are involved in several functional properties of fermented plant products, such as neutralizing free radicals, regulating antioxidant enzyme activities, reducing oxidative stress, affecting inflammatory responses, and enhancing immune system performance [39].

Fermented *Paeoniae alba* extract with plant-derived *Lactobacillus brevis* 174A suppressed inflammatory cytokines interleukin (IL)—6, tumor necrosis factor-α (TNF-α), while simultaneously downregulating the gene expressions of inducible nitric oxide synthase (iNOS), IL-6, TNF-α, and IL-1 compared to the unfermented extract [32]. Fermented *Mercurialis perennis* extract with *Lactobacteria* (*Lactobacillus plantarum* and *Pediococcus pentosaceus*) and non-fermented extract enhanced NFκB and cytokine expression (IL-6, TNF, IL-8, and IL-1) in NFκB-THP-1 reporter cells, showing a concentration-dependent immunostimulatory effect [31]. Fermented *Chenopodium formosanum* leaf extract with *Aspergillus oryzae* increases anti-inflammatory activity via reduced nitric oxide (NO), IL-6, and TNF-α production in lipopolysaccharide (LPS)-stimulated RAW264.7 cells in a dose-dependent manner [23]. It has been found that the fermentation of *Artemisia princeps* plant extract with plant-derived *Lactobacillus plantarum* SN13T generates catechol and secotanapartholide C as IL-8 inhibitors [19]. Fermented maca root extracts with *Lactobacillus* strains, such as *Lactobacillus plantarum, Lactobacillus rhamnosus, Lactobacillus casei*, and *Lactobacillus gasseri*, exhibit higher anti-inflammatory activity than the non-fermented extracts at concentrations of 5% and 10% [29]. Fermented *Laminaria japonica* extract with *Saccharomyces cerevisiae* has stronger anti-inflammatory activity compared to unfermented *Laminaria japonica* extract [26]. Moreover, fermented *Laminaria japonica* extract inhibits the gene expression levels of pro-inflammatory factors (ILs, TNF-α, matrix metallopeptidase 9 (MMP-9)) and activates the nuclear factor 2-related factor 2 (Nrf2) signaling pathway in human immortalized epidermal keratinocytes (HaCaT) exposed to UVB radiation. Fermented *Angelica tenuissima* with *Aspergillus oryzae* was able to play a role in the attenuation of inflammatory responses caused by UVB irradiation via the upregulation of photo-protective hemeoxygease-1 and suppression of proinflammatory COX-2 expression [18].

### 3.4. Melanogenic Inhibitory Effects

**Figure 2:**
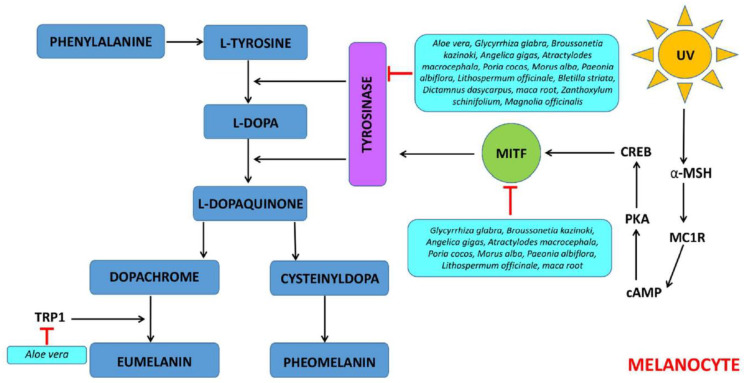
Mechanism for inhibitory effect of FPE on melanogenesis. Legends: αMSH—α-melanocyte-stimulating hormone; MC1R—melanocortin 1 receptor; cAMP—cyclic adenosine monophosphate; PKA—protein kinase A; CREB—cAMP response element-binding protein; MITF—melanocyte-inducing transcription factor; TRP1—tyrosinase-related protein 1.

Skin pigmentation results from several processes, such as melanin synthesis, transport, and accumulation of melanin in keratinocytes [40]. Melanin is a pigment that plays an important role in providing coloration and protecting human skin from the harmful effects of UV light radiation [41]. Some studies showed that FPEs may be inhibitors of melanogenesis and skin-whitening compounds in cosmetics and dermatology (Figure 2).

The fermented leaf skin of *Aloe vera* extract with *Lactobacillus plantarum* BN41 isolated from kimchi may be a natural ingredient that effectively inhibits skin melanogenesis [17]. It was shown that inhibition of tyrosinase activities and melanin synthesis at 0.3% (*w*/*v*) optimal dosage of fermented *Aloe vera* extract was much better than those of arbutin and aloesin, which are commercial skin-lightening ingredients. It was also proved that fermented *Aloe vera* extract effectively downregulated all microphthalmia-associated transcription factors (MITF), tyrosinase-related protein-1 (TYRP-1) and TYRP-2, and tyrosinase (TYR) gene expression, proposing melanogenesis inhibitory mechanism in the MITF/TYRP-1/TYRP-2/TYR pathway. The mixtures of fermented *Glycyrrhiza glabra, Broussonetia kazinoki, Angelica gigas, Atractylodes macrocephala, Poria cocos, Morus alba* (root bark), *Paeonia albiflora*, and *Lithospermum officinale* (2% each) with *Phellinus linteus* showed anti-melanogenic activity in tested cultured B16F0 mouse melanoma cells [24]. Mixtures of fermented plant extract inhibit melanogenesis through the activation of the phosphatidylinositol 3-kinase/Akt/glycogen synthase kinase-3beta signaling pathway and down-regulation of MITF. Moreover, mixtures of fermented plant extract in a dose-dependently manner inhibited melanin and tyrosinase activity and reduced melanogenesis-related proteins, including tyrosinase and MITF in B16F0 cells. The mixture of eight FPEs with *Phellinus linteus* (previously named 8-HsPLCB) showed a reduction in the melanin pigment in melanocytes and histological changes induced by UV irradiation in brown guinea pigs [42]. Moreover, the skin-lightening effect was comparable to arbutin, one of the most widely used ingredients in skin-whitening cosmetics. Fermented mixtures of *Atractylodes macrocephala, Paeonia lactiflora, Bletilla striata, Poria cocos, Dictamnus dasycarpus, Ampelopsis japonica* and *Tribulus terrestris* extract (FB-ChiBai) with *Lactobacillus rhamnosus* at concentrations ranging from 0.05% to 0.5% suppressed the CREB/MITF/tyrosinase melanogenic pathway without inducing cytotoxicity in B16F0 melanoma cells under α-MSH stimulation [20]. Furthermore, it was found that FB-ChiBai significantly attenuated melanin production, tyrosinase activities, and melanogenesis-related signaling pathways and reduced the nuclear translocation and promoter binding activities of MITF in B16F0 murine melanoma cells. Fermented maca root extracts with *Lactobacillus* strains, such as *Lactobacillus plantarum, Lactobacillus rhamnosus, Lactobacillus casei*, and *Lactobacillus gasseri* inhibiting tyrosinase activity, melanin synthesis, and melanogenesis by suppressing MITF-related mechanisms [29]. The fermented *Zanthoxylum schinifolium* extract with *Lactobacillus rhamnosus* A6-5 can be used as an ideal skin whitening agent via greater tyrosinase inhibitory activity and reduced melanin production compared with the raw extract [36]. Fermented *Magnolia officinalis* extracts with *Aspergillus niger* showed higher tyrosinase inhibitory activity than that of the unfermented extracts and the positive control, arbutin, but lower than that of kojic acid [30]. The fermented extracts showed higher inhibitory activity than non-fermented extracts, which may be owed, at least in part, to the combined effects of plant- and bacteria-derived active ingredients (e.g., kojic acid is naturally derived metabolites from *Aspergillus* sp.) [43].

### 3.5. Wound Healing Activity

Wound healing is a complex inter-related biological process at the molecular level, and it occurs in four stages or phases consisting of hemostasis, inflammation, proliferation, and finally epithelialization [44]. Herbal products and their active constituents through different mechanisms of action, including antimicrobial, anti-inflammatory, and antioxidant activity, the stimulation of angiogenesis, the production of cytokines and growth factors, keratinocytes, and fibroblast migration and proliferation, may be considered as an important support during conventional therapy or even as a substitute for synthetic drugs used for wounds treatment [45]. Some research found that FPEs may stimulate wound closure. Fermented hot-water *Trapa japonica* fruit extract with *Bacillus methulotrophicus* and *Bacillus subtilis* stimulate human dermal fibroblast (HDF) and keratinocytes (HaCaT) cells proliferation, and collagen synthesis via activating TGF-β1/GSK-3β/β-catenin pathway [35].

## 4. Fermented Plant Extracts for Dermal Applications

**Figure 3:**
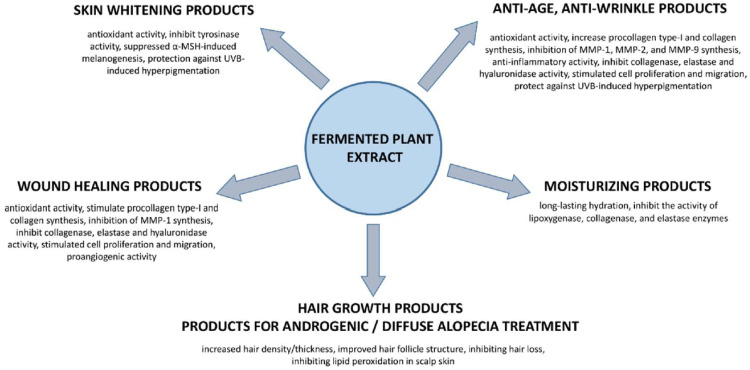
FPE for dermal applications.

The cosmetics and pharmaceutical industries are looking for new products or improvements of existing products with innovative active principles. Herbal products and their active constituents are consistently popular with consumers. Among them are fermented plant products, especially popular in Asian countries and nowadays more noticed in the world markets. FPEs are a rich source of phytochemicals with different biological activities, which can be used as active ingredients in many pharmaceutical/cosmeceutical products [2,46]. Due to a wide spectrum of biological properties, FPEs could be used in several dermal applications like anti-aging and anti-photoaging, anti-wrinkle, skin whitening, moisturizing products, hair growth products, products for androgenic or diffuse alopecia treatment, and wound healing products (Figure 3). Most of them are based on in vitro studies (Table 2). Only a few have been transferred to animal models or clinical trials (Table 3).

### 4.1. Anti-Aging Products

Skin aging is a complex biological process that is influenced by a combination of endogenous (genetics, cellular metabolism, hormones, and metabolic processes) and exogenous (chronic light exposure, pollution, ionizing radiation, chemicals, and poisons) factors [59]. Some research found that fermented plant products affect exogenous factors and pose anti-aging and anti-wrinkle activities.

Mixed root extracts of *Taraxacum officinale* rhizome/root, *Arctium lappa, Anemarrhena asphodeloides, Pueraria lobata*, and *Nelumbo nucifera* fermented with *Saccharomyces cerevisiae* enable human keratinocytes (HEKa) and fibroblasts (HDF) cells proliferation and migration, have anti-aging effects and can be used as active cosmetic raw materials [55]. Fermented *Smilax china* leaves extract with mixtures of *Lactobacillus bulgaricus* and *Lactobacillus reuteri* showed anti-pollution potential through their antioxidant activity and inhibited PGE2 production in HaCaT [34]. *Saccharomyces cerevisiae*-mediated fermented black ginseng has been reported for anti-wrinkle activity in cultured human fibroblasts (HS68) [50]. Moreover, fermented black ginseng increased the expression of type I procollagen and tissue inhibitor of MMP-2 and reduced the expression of MMP-1, MMP-2, and MMP-9 in HS68 cells. Fermented *Magnolia officinalis* extracts with *Aspergillus niger* inhibited skin aging-related enzymes such as collagenase, elastase, MMP-1, and MMP-2 [30]. Moreover, methanol-extracted *M. officinalis* fermented by *A. niger* for 72 h has the most active skincare or antiaging compounds for dermatological applications. Fermented leaves and branches of honeybush extracts indicate a high potential for anti-aging products use through strong antioxidant activity, significant ability to inhibit collagenase and hyaluronidase, and a weak influence on elastase activity, as well as medium photoprotection (sun protection factor, SPF) [53]. *Artemisia vulgaris* fermented solvent fraction showed anti-aging and anti-wrinkle effects via increased collagen synthesis and cell regeneration [49]. The fermented outer layers of the leaf skin of *Aloe barbadensis* at a concentration of 0.3% effectively scavenge cellular ROS generated from the oxidative stress of mitochondria, which results in the inhibition of skin wrinkling processes by increasing collagen production and decreasing MMP-1 production [48]. The fermented *Triticum aestivum, Avena sativa, Helianthus tuberosus, Glycine max*, and *Smallanthus sonchifolius* with *Lactobacillus buchneri* found in kimchi could be potential candidates for the protective effects against UVB-induced photoaging useful natural components of dermatological products [58]. Moreover, a mixture of fermented plant extracts decreased elastase and collagenase activity and increased type I collagen expression and MMP mRNA levels in UVB-induced photoaging of normal human dermal fibroblasts and epidermal keratinocytes. Furthermore, FPEs promoted the expression of moisture factor and anti-oxidant enzymes in UVB-induced photoaging in vitro models. Also, *Lavandula angustifolia* extract fermented with *Pediococcus pentosaceus* DK1 showed an MMP-1 expression lower than that in UVB-irradiated fibroblasts treated with non-fermented extract [27]. Moreover, fibroblasts treated with fermented *L. angustifolia* extract showed 20% less reduction in collagen production upon UVB irradiation than those treated with non-fermented extract. The fermented *Angelica tenuissima* root with *Aspergillus oryzae* showed anti-photoaging potential and could be utilized as an effective ingredient in anti-aging and anti-wrinkle products [18]. Fermented *Angelica tenuissima* was able to improve extracellular matrix impairment caused by UVB irradiation through the upregulation of procollagen type-1 synthesis and secretion as well as the suppression of MMP-1 and elastase expression in HaCaT (human keratinocyte) or Hs68 (human foreskin fibroblast) skin cells. The aqueous extract of *Fructus arctii* was fermented with *Grifola frondosa* UV-A exposed human dermal fibroblasts, showing reduced expressions of MMP-1 and collagen biosynthesis [52]. *Citrus unshiu* peel aqueous extracts were fermented by *Schizophyllum commune*, reducing the expression of MMP-1 and collagen biosynthetic activity in a dose-dependent manner after UV-A exposed human dermal fibroblasts [51]. The fermented *Aloe arborescens* extract with *Lactobacillus plantarum* enhanced anti-skin wrinkling due to synergistic effects between the barbaloin and the low-molecular-weight polysaccharides retained after the fermentation process [47].

The fermented *Trapa japonica* fruit extract stimulated the synthesis of collagen, reduced TNF-α-induced gene expression of MMPs in human dermal fibroblast cells, and promoted wound recovery in HaCaT cells [56]. Moreover, a randomized and double-blind clinical trial showed that eye cream with 0.5% peptide isolated from fermented *Trapa japonica* extract application on the eye twice a day for 8 weeks by participants (22 healthy women aged 41 to 57 years) significantly reduced skin wrinkles.

### 4.2. Skin Whitening Products

The fermented *Aloe vera* extract with *Lactobacillus plantarum* BN41 in a concentration of 0.3% (*w*/*v*) can be a natural ingredient with fewer side effects for replacement of many synthetic and chemical skin-lightening components in pharmaceutical and dermatological products [17]. The fermented mixtures of *Atractylodes macrocephala, Paeonia lactiflora, Bletilla striata, Poria cocos, Dictamnus dasycarpus, Ampelopsis japonica*, and *Tribulus terrestris* extract (FB-ChiBai) with *Lactobacillus rhamnosus* can protect against UV-B irradiation and that it might be used as an agent in products to protect against UVB-induced hyperpigmentation [20]. In the in vivo experiments, FB-ChiBai was topically applied to the dorsal skin of C57BL/6J nude mice and concurrently irradiated with UVB three times a week for 8 weeks. The results indicated that FB-ChiBai alleviated UVB-induced hyperpigmentation by reducing epidermal hyperplasia and inhibiting the CREB/MITF/tyrosinase pathway.

### 4.3. The Moisturizing Products

The fermented Yerba Mate extract with Kombucha showed antioxidant activity, a strong ability to inhibit collagenase and elastase enzymes in vitro study, and long-lasting hydration and reduced transepidermal water loss (TEWL) after application on the volunteers’ forearm skin (0.2 mL of 100 µg/mL FPE) in in vivo study [7].

### 4.4. The Hair Growth Products

*Bacillus/Trapa japonica* fruit ferment filtrate extracts (TJFs) enhance human hair follicle dermal papilla (HDP) cell proliferation and migration via the Akt/ERK/GSK-3β signaling pathway, suggesting a potential treatment for alopecia [57]. The TJFs also induced cell cycle progression, inhibited type I 5α-reductase, decreased apoptosis, and enhanced angiogenesis via increased vascular endothelial growth factor (VEGF) and vascular expansion in CAM assay. Moreover, insulin-like growth factor-1 and keratinocyte growth factor, stimulating hair growth were detected in the human dermal papilla. Also, shampoo and lotion for hair care containing fermented papaya, fermented mangosteen, and caffeine were applied to 154 subjects of both sexes with clinically confirmed androgenic or diffuse alopecia for 3 months in a randomized double-blind clinical trial [54]. The hair care products significantly inhibited hair loss, increased hair density/thickness, and improved hair follicle structure versus placebo and caffeine controls. The products with fermented papaya and fermented mangosteen substantially normalized the microbiota pattern and increased ATP content in hair follicles, while inhibiting lipid peroxidation in the scalp skin, and SH-group formation in the hair shaft.

### 4.5. Wound Healing Products

Fermented *Carica papaya* preparation after 8 weeks of oral supplementation (0.2 g/kg body weight) improved wound healing activity in adult obese diabetic (db/db) mice [22]. Diabetic mice supplemented with fermented papaya showed a higher abundance of CD68 as well as CD31 at the wound site, suggesting effective recruitment of monocytes and an improved proangiogenic response.

## 5. Toxicological Aspect

Fermented plants have a long history of safe human consumption, while research into other beneficial effects of bioferments in skin care products and pharmaceuticals is still ongoing. Some research found that fermentation increased the biological effectiveness and bioavailability of fermented plants and decreased cytotoxicity compared to non-fermented plants [2]. The significant increase in cell viability of fibroblasts and keratinocytes for Yerba Mate ferments was observed in 500–1000 μL/mL, while Yerba Mate extracts at the highest concentration used (1000 µg/mL) caused a cytotoxic effect on fibroblasts [7]. The viability of a HaCaT cell was not reduced during treatment with 0.125–1% of fermented *Smilax china* leaves extract, but the viability was significantly reduced from 2% of FPE [34]. Fermented *Zanthoxylum schinifolium* extracts (<500 mg/mL) have no cytotoxic effect [36]. However, cell viability fermented *Zanthoxylum schinifolium* extracts beyond the concentration of 500 mg/mL dropped from 100% to 89.98% [36]. *Aloe vera* fermented extract showed no cytotoxicity against murine melanoma cells at concentrations below 0.5% (*w*/*v*) [17]. Fermented *Angelica tenuissima* (125–1000 μg/mL) treatment did not show any significant cytotoxic or cell proliferative effect on Hs68 or HaCaT cells [18]. Fermentation plant mixtures FB-ChiBai were found to have no cytotoxic effect on B16F0 cells at a concentration of 0.5% [20]. Fermented *Chenopodium formosanum* leaf extract (≤400 mg/L) was not cytotoxic [23]. Also, fermented *Aloe arborescens* [47] and *Artemisia vulgaris* [49] showed no cytotoxicity in the MTT test.

*Laminaria japonica* fermented freeze-dried powder is non-irritating to the eyes, has high safety, and can be added to skin care products as a functional raw material [26]. The aqueous and Lactobacterial-fermented *Mercurialis perennis* extracts were tested for micronuclei formation in THP-1 cells and toxicity in luminescent bacteria (*V. fischeri*), whereby no mutagenic or toxic effects were detected, which corroborates their safe use in pharmaceutical remedies [31]. Unfortunately, most of the studies presented in this paper do not include toxicological tests, which should be crucial for FPE and their use in cosmetics and drugs applied topically to the skin.

## 6. Future Research Directions

Fermentation could be considered a significant technique to obtain bioactive compounds with a broad spectrum of structural diversity and different biological activities useful for dermal applications. Fermentation is a feasible strategy for enhancing the bioactivity of herbal medicines via breaking down or converting the undesirable substrates into compatible components under the action of microbial enzymes, thereby improving the substrate properties via the production and enrichment of bioactive compounds [60]. Fermentation significantly increases the phenolic and anthocyanin contents, which reveals stronger antioxidant activities of FPE compared to non-fermented plants [9,11,26,27,33]. Bacteria and fungi have great potential for the production of antioxidants through enzymatic hydrolysis of phenolic glycosides to free polyphenols [61]. Polyphenols also enhance antimicrobial [62], anti-inflammatory [63] and wound healing activities [64]. Unfortunately, although FPE showed many desirable biological activities, their practical use in the cosmetic and pharmaceutical industries is negligible. Technological development of the fermentation process requires not only the appropriate selection of plants and starter cultures of microorganisms but also the optimization of fermentation conditions (temperature, pH, time of fermentation, etc.) which will collectively affect the presence of active compounds and their biological activities. Moreover, there are some important problems in the production of FPE, such as the possible generation of methanol, formaldehyde, biogenic amines, and nitrite during fermentation, as well as storage stability [65]. Also, the determination of the phytochemical composition of fermented plant extract brings many difficulties. The extraction of bioactive compounds from fermented plants and their quantitative and qualitative estimation is important for the exploration of new biocompounds to be used by the pharmaceutical industry both directly and/or indirectly as lead molecules used to synthesize more potent molecules. The isolation, purification, and identification of compounds responsible for the biological activity remain a great challenge in the drug discovery process. The various techniques involving the applications of chromatographic techniques such as HPLC (High-Performance Liquid Chromatography), TLC (Thin Layer Chromatography), HPTLC (High-Performance Thin Layer Chromatography), OPLC (Optimum Performance Laminar Chromatography), GC (Gas Chromatography), PC (Paper Chromatography), CC (Column Chromatography) and detection through Fourier Transform Infra-Red spectroscopy (FTIR), Nuclear Magnetic Resonance (NMR), and Mass Spectrometry (MS) offers enormous possibilities, but require experience in working with plant material and specialist knowledge in operating the above devices [66]. The selection of the isolation and identification methods depends on the properties of the bioactive substance and is a difficult and time-consuming process [65]. Therefore, most research on fermented plants is limited to determining the group of compounds found in the sample (e.g., polysaccharides, polyphenols, flavonoids). Most of the described research is conducted on cell lines in vitro methods (Table 2) and only concerns potential dermal applications. Little research has been transferred to animal models or clinical trials (Table 3), which are expensive and require appropriate permits. Fermented plant products have many valuable biological activities that could be used by the cosmetic and pharmaceutical industries but require further research.

## 7. Conclusions

FPE are innovative ingredients formed from plant raw materials during the fermentation process with appropriate strains of microorganisms. FPE showed different biological activities, including antimicrobial, antioxidant, anti-inflammatory, anti-melanogenic, and wound healing activity. Their biological activities may be used for several dermatological applications, including anti-aging and anti-photoaging products, skin whitening products, moisturizing products, hair growth products, products for androgenic or diffuse alopecia treatment, and wound healing products.

## Figures and Tables

**Table 1:** Fermented plant extract and their biological activity.

Plants	Microorganism	Active Compounds in FPE	Biological Activity	Ref.
*Aloe vera*	*Lactobacillus plantarum* BN41	aloesin	melanogenic inhibitor	[17]
*Angelica tenuissima*	*Aspergillus oryzae*	-	anti-inflammatory activity	[18]
*Artemisia princeps*	*Lactobacillus plantarum* SN13T	catechol, seco-tanapartholide C	anti-inflammatory activity	[19]
*Atractylodes macrocephala, Paeonia lactiflora, Bletilla striata, Poria cocos, Dictamnus dasycarpus, Ampelopsis japonica, Tribulus terrestris*	*Lactobacillus rhamnosus*	-	melanogenic inhibitor	[20]
*Vaccinium corymbosum* (blueberry fruit)	*Bacillus amyloliquefaciens, Starmerella bombicola, Lactobacillus brevis*	-	antibacterial activity antioxidant activity	[21]
*Carica papaya*	-	-	wound healing activity	[22]
*Chenopodium formosanum*	*Aspergillus oryzae*	protocatechuic acid, epicatechin, gallic acid, quercetin	antioxidant activity anti-inflammatory activity antimicrobial activity skin-whitening activity	[23]
ginseng	*Aspergillus usamii*	-	antioxidant activity	[10]
*Glycyrrhiza glabra, Broussonetia kazinoki, Morus alba, Angelica gigas, Atractylodes macrocephala, Poria cocos, Paeonia albiflora, Lithospermum officinale*	*Phellinus linteus*	-	melanogenic inhibitor	[24]
green coffee beans	kombucha	caffeine, trigonelline, phenolic compounds	antioxidant activity	[25]
*Laminaria japonica*	*Saccharomyces cerevisiae*	polysaccharide, phenolic compounds	antioxidant activity anti-inflammatory activity	[26]
*Lavandula angustifolia*	*Pediococcus pentosaceus* DK1	luteolin-7-O-glucoside, apigenin-7-O-glucoside, chlorogenic acid	antioxidant activity	[27]
Lupin (*Lupinus polyphyllus*)	*Aspergillus oryzae*	selenium nanoparticles	antimicrobial activity	[28]
maca root	*Lactobacillus plantarum, Lactobacillus rhamnosus Lactobacillus casei, Lactobacillus gasseri*	polyphenols	anti-inflammatory activity melanogenic inhibitor	[29]
*Magnolia denudata*	*Pediococcus acidilactici* KCCM 11614	polyphenols	antioxidant activity	[9]
*Magnolia officinalis*	*Aspergillus niger*	-	antibacterial activity melanogenic inhibitor	[30]
*Mercurialis perennis*	*Lactobacillus plantarum* *Pediococcus pentosaceus*	cinnamic acids depsides containing glucaric, malic, and 2-hydroxyglutaric acids along with quercetin and kaempferol glycosides	anti-inflammatory activity	[31]
*Paeoniae alba*	*Lactobacillus brevis* 174A	pyrogallol	antioxidant activity anti-inflammatory activity	[32]
*Panax notoginseng*	*Saccharomyces cerevisiae* CGMCC 17452	polysaccharide, ginsenoside, flavonoids	antioxidant activity	[33]
*Rhus verniciflua*	-	-	antioxidant activity	[11]
*Smilax china*	*Lactobacillus bulgaricus, Lactobacillus reuteri*	-	antioxidant activity	[34]
*Trapa japonica*	*Bacillus methulotrophicus* *Bacillus subtilis*	-	wound healing activity	[35]
*Zanthoxylum schinifolium*	*Lactobacillus rhamnosus* A6-5	benzamides, ginsenoside, tricosanamide, gynuramide	antibacterial activity melanogenic inhibitor	[36]

**Table 2:** Fermented plant extract and their potential dermal applications—in vitro study.

Plants	Microorganism	Active Compounds	Biological Activity	Potential Application	Ref.
*Aloe arborescens*	*Lactobacillus plantarum*	barbaloin polysaccharides	antioxidant activity collagen production inhibition of MMP-1 synthesis	anti-wrinkle product	[47]
*Aloe barbadensis*	*Lactobacillus plantarum*	quercetin	antioxidant activity collagen production inhibition of MMP-1 synthesis	anti-wrinkle product protection against oxidative stress	[48]
*Aloe vera*	*Lactobacillus plantarum* BN41	aloesin	antioxidant activity inhibition of tyrosinase inhibition of melanin synthesis	skin whitening product	[17]
*Angelica tenuissima*	*Aspergillus oryzae*	-	increase wound healing stimulate procollagen type-I and elastase synthesis inhibition of MMP-1 synthesis	anti-photoaging product	[18]
*Artemisia vulgaris*	*Bacillus methanolicus* *Bacillus subtilis*	-	increase wound healing stimulate procollagen type-I and collagen synthesis inhibition of MMP-1 synthesis	anti-aging product	[49]
*Atractylodes macrocephala, Paeonia lactiflora, Bletilla striata, Poria cocos, Dictamnus dasycarpus, Ampelopsis japonica, Tribulus terrestris*	*Lactobacillus rhamnosus*	-	suppressed α-MSH-induced melanogenesis significantly attenuated melanin production and tyrosinase activities	skin whitening product	[20]
black ginseng	*Saccharomyces cerevisiae*	-	stimulate type I procollagen synthesis decrease MMP-1, MMP-2 and MMP-9 synthesis increase TIMP-2 expression	anti-wrinkle product	[50]
*Citrus unshiu*	*Schizophyllum commune*	hesperetin	decrease in the expression level of MMP-1 increase collagen synthesis	anti-photoaging product	[51]
*Fructus arctii*	*Grifola frondosa*	arctigenin caffeic acid	antioxidant activity anti-inflammatory activity inhibition of MMP-1 activity	anti-aging product	[52]
honeybush	*-*	mangiferin hesperidin	antioxidant activity inhibit collagenase, tyrosinase, and hyaluronidase activity	anti-aging product wound healing product	[53]
*papaya, mangosteen*	*-*	-	increased hair density improved hair follicle structure inhibiting hair loss inhibiting lipid peroxidation in scalp skin	lotion for androgenic or diffuse alopecia	[54]
*Taraxacum officinale, Arctium lappa, Pueraria lobata, Anemarrhena asphodeloides, Nelumbo nucifera*	*Saccharomyces cerevisiae*	-	antioxidant activity anti-inflammatory activity	anti-aging product	[55]
*Trapa japonica*	*Bacillus subtilis* *Bacillus methylotrophicus*	-	increase collagen synthesis reduction expression levels of MMP-1 and MMP-9	anti-aging product	[56]
*Trapa japonica*	*Bacillus subtilis* *Bacillus methylotrophicus*	-	stimulated cell proliferation and migration inhibited type I 5α-reductase enhanced angiogenesis	hair growth products treatment for alopecia	[57]
*Triticum aestivum, Avena sativa, Glycine max, Helianthus tuberosus, Smallanthus sonchifolius*	*Lactobacillus buchneri*	-	antioxidant activity decreased elastase activity increased type I collagen expression in a UVB-induced fibroblast and keratinocytes	anti-photoaging product	[58]
*Yerba Mate*	Kombucha	caffeoylquinic acid, dicaffeoylquinic acid	antioxidant activity inhibit the activity of lipoxygenase, collagenase, and elastase enzymes	moisturizing product	[7]

Legends: MMP-1—matrix metalloproteinase-1; α-MSH—alpha-melanocyte stimulating hormone; TIMP-2—metalloproteinase-2.

**Table 3:** Fermented plant extract and their dermal applications—in vivo study.

Plants/Active Compound	Microorganism	Effective Dose of FPE vs. Control	Research Model	Application	Ref.
*A. macrocephala, P. lactiflora, B. striata, P. cocos, D. dasycarpus, A. japonica, T. terrestris*	*Lactobacillus rhamnosus*	vehicle with 2% and 6% FPE control: vehicle treatment: 3 times a week for 8 weeks	C57BL/6J nude mice	skin whitening product (anti-melanogenic effects, protection against UVB-induced hyperpigmentation)	[20]
*Carica papaya*	*-*	oral supplementation (0.2 g/kg) FPE control: placebo supplement treatment: 5 days/week for 8 weeks	obese diabetic (db/db) mice	diabetic wound healing product (proangiogenic activity)	[22]
*papaya, mangosteen*	*-*	placebo group (*n* = 29), experimental group (*n* = 100)-lotion with 2.0% *w*/*w* for each FPE, control group (*n* = 25)-lotion with 0.5% *w*/*w* caffeine treatment: lotion-daily once a day, shampoo-3 times a week for 3 months	154 women and men with androgenic/diffuse alopecia	product for androgenic or diffuse alopecia (hair care cosmetics significantly inhibited hair loss, increased hair density/thickness, and improved hair follicle structure)	[54]
*Trapa japonica*	*Bacillus subtilis* *B. methylotrophicus*	eye cream with 0.5% peptide isolated from FPE/control: none treatment: twice a day for 8 weeks	22 women, aged 41 to 57 years	anti-aging product (ant wrinkle activity)	[55]
*Yerba Mate*/caffeoylquinic acid, dicaffeoylquinic acid	Kombucha	0.2 mL of 100 µg/mL fermented yerba mate control: none	15 volunteers	moisturizing and long-lasting hydration product (inhibit the activity of lipoxygenase, collagenase, and elastase enzymes)	[7]
